# Is women’s empowerment associated with coverage of RMNCH interventions in low- and middle-income countries? An analysis using a survey-based empowerment indicator, the SWPER

**DOI:** 10.7189/jogh.11.04015

**Published:** 2021-03-01

**Authors:** Fernanda Ewerling, Fernando C Wehrmeister, Cesar G Victora, Anita Raj, Lotus McDougal, Aluisio JD Barros

**Affiliations:** 1 International Center for Equity in Health, Federal University of Pelotas, Pelotas, Brazil; 2 Postgraduate Program in Epidemiology, Federal University of Pelotas, Pelotas, Brazil; 3 Center on Gender Equity and Health, University of California San Diego, San Diego, California, USA

## Abstract

**Background:**

Women’s empowerment has a strong potential to promote sustainable development. We evaluate the association between women’s empowerment and the Composite Coverage Index (CCI), a weighted average of coverage of eight interventions in reproductive, maternal, newborn and child health (RMNCH). We also assess whether these effects are modified by wealth.

**Methods:**

We used Demographic and Health Survey data from 62 low- and middle-income countries. Women’s empowerment was measured using the three domains (attitude to violence, social independence and decision making) of the survey-based indicator of women’s empowerment (SWPER). Analyses followed an ecological design. Meta-regression models were used to account for within-country uncertainty in the CCI. We also carried out meta-regression with wealth quintiles of households as the units of analyses and tested for interaction between wealth and each empowerment domain.

**Results:**

We found positive associations between the three domains of SWPER and CCI at the country level. One standard deviation change in empowerment increased the CCI by 14.2 percentage points (attitude to violence), 15.3 percentage points (decision-making), and 16.3 percentage points (social independence). The association between social independence and CCI was modified by wealth: each additional standard deviation was associated with 21.8 (95% confidence interval (CI) = 14.0-29.6) and 8.7 (95% CI = 5.4-12.0) percentage points increase in the CCI among the poorest and the richest quintiles, respectively.

**Conclusions:**

Our findings suggest that efforts toward the achievement of SDG5 (Achieve gender equality and empower all women and girls) may support improvements in RMNCH in low- and middle-income countries, especially among the poorest women and children.

In 2015, the United Nations launched a new set of objectives to guide countries towards sustainable development. Given its potential to promote economic growth, reduce poverty and accomplish human rights, the fifth Sustainable Development Goal (SDG) specifically mentions the need to “empower all women and girls”. Women’s empowerment is a complex concept often defined as an increased capacity to make purposive choices and to transform those choices into desired actions and outcomes [1]. More empowered women are more likely to provide better care for their children (immunization and nutrition) [2-4] and have higher levels of health service utilization both for themselves and for their children, including institutional delivery, antenatal care and family planning [4-6], thus also contributing to the third SDG on health.

The Countdown to 2030 is a monitoring and accountability initiative aimed at assessing country progress towards the SDGs. Countdown has, since its inception, focused on monitoring health intervention coverage in low and middle-income countries, with special attention to reproductive, maternal, newborn and child health (RMNCH). A set of essential, proven interventions has been selected for this monitoring exercise. Such interventions, like contraception, antenatal care, postnatal care, immunization, cover the whole RMNCH continuum of care [7,8]. One of the Countdown innovations is the Composite Coverage Index (CCI), a summary measure of universal health coverage in reproductive, maternal, newborn and child health (RMNCH) based on eight preventive and curative interventions related to family planning, maternity care, child immunization and illness management [7,9]. The CCI is a group-level rather than an individual-level measure, though it may be calculated at national level or for subgroups such as wealth quintiles or geographical regions. It has been used in many papers published previously [8-10] and in reports, including WHO and UNICEF reports [11,12]. The CCI tends to document inequality patterns more precisely than standalone coverage indicators, and can also be used to predict levels of child mortality and undernutrition [8].

Global coverage with RMNCH interventions, such as demand for family planning satisfied with modern methods, skilled birth attendance, children vaccination, among others has been increasing steadily [13,14], but progress is uneven and major gaps persist both between and within countries [15,16]. While RMNCH intervention coverage has substantial variability across social strata, the association with women’s empowerment is less understood.

Understanding the relationship between women’s empowerment and RMNCH intervention coverage can inform the development of appropriate policies to reach all women and children. In 2017, a novel Survey-based Women’s emPowERment (SWPER) indicator was proposed for African countries [4] and, in 2019, an updated and more comprehensive version – the SWPER global – was developed, allowing the assessment of women’s empowerment levels within low- and middle-income countries (LMICs) from all world regions [17].

Examination of women’s empowerment requires consideration of social equity, as social and gender inequities intersect, leaving socially marginalized women and girls most vulnerable to poor health outcomes [18]. Recognizing the intersectionality of inequalities, it is plausible that social equity indicators such as household wealth may modify observed associations between women’s empowerment and health intervention coverage [19]. We hypothesize that the associations between empowerment and positive health outcomes may be enhanced in higher household wealth contexts and attenuated in lower household wealth contexts. If a significant effect modification exists, these results would indicate the promise of gender equity-focused programs to improve health across socioeconomic strata.

The objective of this manuscript is to assess the relationship between women’s empowerment and RMNCH intervention coverage. Specifically, we will assess which domains of women’s empowerment are most associated with RMNCH intervention coverage, and whether these effects are moderated by household wealth, with a goal of informing program and policy decisions. This will be accomplished using the SWPER index as a measure of empowerment (focusing on the three empowerment domains - attitude to violence, social independence and decision making) and the CCI as a measure of RMNCH intervention coverage across 62 LMICs representing seven world regions.

## METHODS

We used publicly available data sets from Demographic and Health Surveys (DHS) with available information to estimate the SWPER and the CCI. For each LMIC, we included the latest available survey. In total, we analyzed 62 LMICs, with survey years ranging from 2001 to 2017. The DHS are a series of nationally representative, cross-sectional health surveys conducted in low- and middle-income countries [20]. As all surveys present similar questionnaires, methodology and sampling strategy (multi-stage cluster sampling), their results are comparable across countries. We also considered using other types of national surveys such as Multiple Indicator Cluster Surveys, but their questionnaires did not include all necessary variables.

Women’s empowerment was measured using the SWPER global [17], which is a modified version of the SWPER originally developed for African countries [4]. The differences between the original SWPER and the global version of the index were based on recommendations from experts on women’s empowerment given in a workshop held at the Pan American Health Organization headquarter in partnership with the International Center for Equity in Health in 2018 (details are provided in Table S1 in the **Online Supplementary Document**). The SWPER is an individual-level indicator based on 14 questions from national surveys to assess three empowerment domains: attitude to domestic violence, which comprises questions related to the women’s opinion on whether beating the wife is justified in some situations; social independence, that includes preconditions for empowerment, such as the woman’s access to information, educational attainment, age at first marriage and first child, and difference in age and education between the woman and her husband; and decision making, which is comprised of three questions on who makes decisions in the household in regard to the respondent’s health care, major expenses and to visits to family and relatives. The SWPER is a cross-culturally tested tool that allows the measurement of women’s empowerment among partnered women at individual-level and by subgroups of women. Full details on the construction of the index and its validity are presented elsewhere [17]. The resulting scores are standardized numbers, so positive values represent above-average levels of empowerment, and negative values represent the opposite, with zero being the average value for LMICs.

We used the CCI as a summary indicator for RMNCH intervention coverage. The CCI is an average of eight interventions along the four stages of the continuum of care, with each stage having the same weight [7,13]. Its component indicators are: reproductive health – demand for family planning satisfied with modern methods (DFPSm); maternal and newborn health – at least four antenatal care visits (ANC4) and skilled birth attendance (SBA); immunization – three doses of diphtheria-tetanus-pertussis vaccine (DPT3), measles vaccine (MSL) and Bacillus Calmette-Guérin vaccine (BCG); management of child illness – oral rehydration salts for children with diarrhea (ORS) and care-seeking for children with suspected pneumonia (CPNM) [7]. The definition of each component indicator is provided in Table S2 in the **Online Supplementary Document**. The CCI is calculated by the expression:


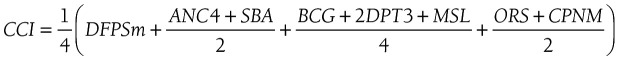


The CCI was calculated at national level and for wealth quintiles. Coverage indicators followed the standard Countdown to 2030 definitions [7]. The wealth index is obtained through principal component analysis based on household assets and characteristics of the dwelling. In the DHS’ publicly available data sets, a value of the index is assigned to each household in the sample and provided with the survey. [21], This wealth index is divided into quintiles, with the first quintile representing the 20% poorest and the fifth the 20% richest households. This is therefore a within-country, relative ranking of comparative household wealth.

Meta-regression models were used to account for the CCI within-country uncertainty, as measured by the standard errors. Meta-regression is an extended version of the variance-weighted least squares regression that considers an additional variance component, which is assumed to be equal across units [22]. Analyses were performed using countries as the unit of analysis and were not weighted by countries’ population size. Analyses were repeated controlling for the log of the Gross Domestic Product (GDP) per capita adjusted by the purchase power parity in international dollars using data provided by the World Bank [23]. To evaluate whether wealth modifies the effect of empowerment, we carried out meta-regression with wealth quintiles as the unit of analysis and tested the interaction between wealth and each domain of the SWPER. Lastly, as mother’s education is known to be strongly related to RMNCH intervention coverage [13], we carried out sensitivity analyses to evaluate the association between CCI and empowerment, after removing the education variable from the SWPER score.

Estimates and respective standard errors accounted for the sample design, including clusters, strata and sample weights. All analyses were conducted using the Stata software (StataCorp. 2017. Stata Statistical Software: Release 15. College Station, TX: StataCorp LP). Ethical clearance was obtained by the national institutions that carried out the surveys. All data sets were anonymized.

## RESULTS

A description of the countries in terms of national CCI and mean women’s empowerment scores in each SWPER domain is presented in **Table 1**. The average CCI coverage considering all countries was 64.9%. There are substantial inequalities across countries, with coverage ranging from 28% in Chad to almost 80% in Dominican Republic and Honduras. 14 out of the 62 countries analyzed have CCI coverage levels below 50%, with eight of these being from West and Central Africa. Countries with positive SWPER values are above the LMIC average [17]. Guinea, Chad, Niger, and Mali presented some of the lowest SWPER scores for the three domains, while Dominican Republic, South Africa and Moldova presented the highest scores and also some of the highest CCI levels (79.6%, 75.2% and 73.4%, respectively). Honduras has the highest CCI (79.7%), as well as relatively high scores for attitude to violence and decision-making. However, its scores for social independence are much smaller, albeit still positive (0.07).

**Table 1:** List of countries included in the analyses, with their world region, ISO-code, year of the survey, Composite Coverage Index (CCI) estimate and mean women’s empowerment level in attitude to violence, social independence and decision making

					Mean women’s empowerment		
**World Region**	**Country**	**Survey year**	**ISO code**	**CCI (%)**	**Attitude to violence**	**Social independence**	**Decision making**
South Asia	Afghanistan	2015	AFG	48.0	-0.78	-0.49	-0.30
	Bangladesh	2014	BGD	65.1	0.36	-0.58	0.13
	India	2015	IND	71.4	-0.03	0.08	0.44
	Maldives	2009	MDV	74.1	0.29	0.40	0.51
	Nepal	2016	NPL	64.7	0.39	-0.23	-0.05
	Pakistan	2017	PAK	61.7	-0.16	0.03	-0.28
East Asia & the Pacific	Cambodia	2014	KHM	69.4	-0.13	0.15	0.87
	Indonesia	2012	IDN	75.2	0.32	0.43	0.68
	Myanmar	2015	MMR	67.5	0.04	0.48	0.59
	Philippines	2017	PHL	70.2	0.63	0.81	0.92
	Timor-Leste	2016	TLS	63.0	-0.95	0.36	0.87
Europe & Central Asia	Albania	2008	ALB	64.2	0.29	0.80	0.53
	Armenia	2015	ARM	73.5	0.59	0.80	0.86
	Azerbaijan	2006	AZE	46.8	-0.20	0.75	0.22
	Kyrgyzstan	2012	KGZ	70.0	0.15	0.83	0.85
	Moldova	2005	MDA	73.4	0.50	0.85	1.12
	Tajikistan	2012	TJK	69.4	-0.61	0.54	0.04
Middle East & North Africa	Egypt	2014	EGY	78.4	0.17	0.29	0.42
	Morocco	2003	MAR	61.4	-0.73	-0.01	-0.08
West & Central Africa	Benin	2011	BEN	51.3	0.37	-0.38	0.03
	Burkina Faso	2010	BFA	54.6	-0.09	-0.61	-0.73
	Cameroon	2011	CMR	47.6	-0.01	-0.33	-0.28
	Chad	2014	TCD	28.0	-0.94	-0.83	-0.58
	Congo Democratic Republic	2013	COD	47.1	-0.68	-0.30	-0.10
	Cote d’Ivoire	2011	CIV	43.6	-0.20	-0.45	-0.51
	Gabon	2012	GAB	58.1	0.06	0.25	0.32
	Gambia	2013	GMB	61.5	-0.33	-0.47	0.06
	Ghana	2014	GHA	65.3	0.21	0.07	0.49
	Guinea	2012	GIN	39.9	-1.53	-0.80	-0.50
	Liberia	2013	LBR	60.3	0.00	-0.46	0.52
	Mali	2012	MLI	45.2	-0.84	-0.61	-1.06
	Niger	2012	NER	45.4	-0.68	-0.87	-0.84
	Nigeria	2013	NGA	37.7	0.02	-0.35	-0.44
	Sao Tome & Principe	2008	STP	68.1	0.43	-0.09	0.27
	Senegal	2017	SEN	61.9	-0.46	-0.20	-0.84
	Sierra Leone	2013	SLE	66.4	-0.64	-0.58	-0.07
	Togo	2013	TGO	52.1	0.16	-0.21	-0.22
Eastern & Southern Africa	Angola	2015	AGO	45.5	0.25	-0.21	0.57
	Burundi	2016	BDI	62.6	-0.40	-0.08	0.33
	Comoros	2012	COM	51.7	0.02	0.03	-0.23
	Eswatini	2006	SWZ	78.1	0.50	0.35	0.15
	Ethiopia	2016	ETH	45.1	-0.68	-0.59	0.52
	Kenya	2014	KEN	70.4	0.10	0.11	0.46
	Lesotho	2014	LSO	75.3	0.32	0.33	0.67
	Madagascar	2008	MDG	49.8	0.27	-0.22	0.74
	Malawi	2015	MWI	77.0	0.49	-0.29	0.24
	Mozambique	2011	MOZ	54.6	0.40	-0.42	0.18
	Namibia	2013	NAM	77.0	0.28	0.84	0.75
	Rwanda	2014	RWA	67.7	0.10	0.31	0.51
	South Africa	2016	ZAF	75.2	0.68	1.00	0.93
	Tanzania	2015	TZA	62.3	-0.43	-0.04	0.04
	Uganda	2016	UGA	65.1	-0.03	-0.20	0.32
	Zambia	2013	ZMB	69.5	-0.20	-0.18	0.39
	Zimbabwe	2015	ZWE	73.1	0.24	0.19	0.76
Latin America & Caribbean	Bolivia	2008	BOL	61.7	0.52	0.34	0.80
	Dominican Republic	2013	DOM	79.6	0.75	0.46	0.91
	Guatemala	2014	GTM	68.8	0.60	0.10	0.64
	Guyana	2009	GUY	71.3	0.52	0.58	0.95
	Haiti	2016	HTI	49.8	0.50	0.31	0.59
	Honduras	2011	HND	79.7	0.56	0.07	0.64
	Nicaragua	2001	NIC	75.3	0.52	-0.05	0.58
	Peru	2016	PER	74.3	0.73	0.78	0.77

ISO – International Organization for Standardization, CCI – Compositive Coverage Index

**Figure 1:**
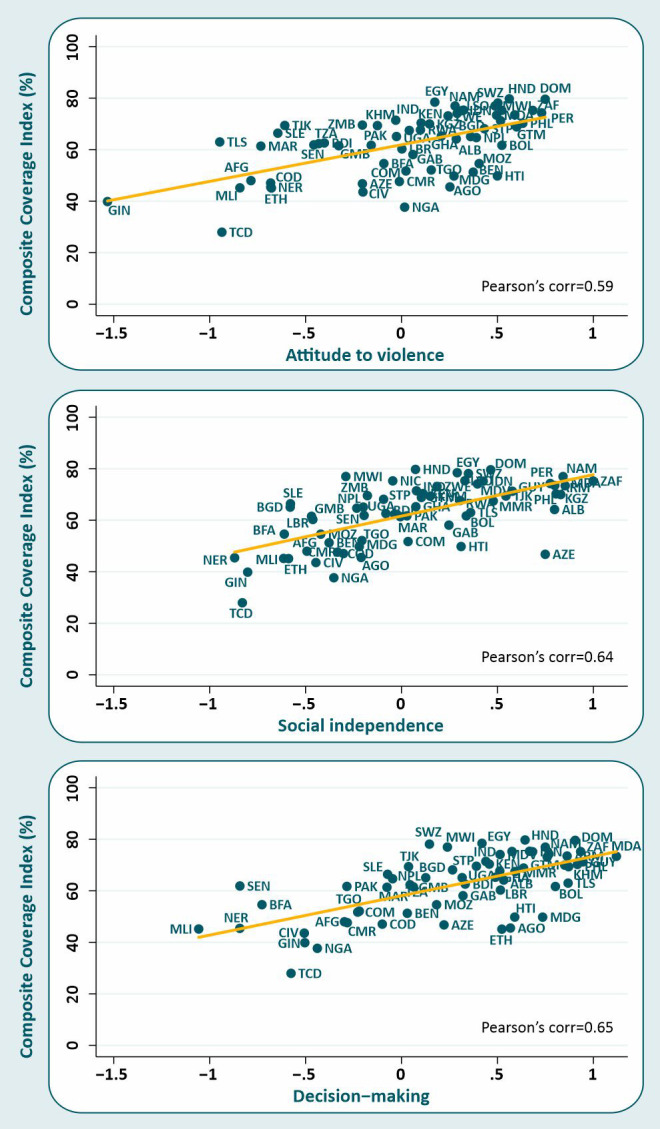
Effect of women’s empowerment for each of the three domains of the SWPER (attitude to violence, social independence and decision making) on the composite coverage index (CCI) at national level.

All three domains of empowerment were positively associated with the CCI. Pearson correlation coefficients were 0.59, 0.64 and 0.65 for attitude to violence, social independence and decision making, respectively (**Figure 1**), with all *P*-values <0.001. The CCI increased by 14.2 (95% confidence interval (CI) = 9.2-19.3), 16.1 (95% CI = 11.1-21.1) and 15.3 (95% CI = 10.7-19.9) percentage points, on average, for each standard deviation increase in attitude to violence, social independence and decision making, respectively. The effects hardly changed after adjustment for the log GDP per capita (**Table 2**). We performed a sensitivity analysis by excluding woman’s education from the social independence SWPER domain, as it could be driving the positive results. Paradoxically, after this exclusion we found a stronger association between the social independence and the CCI (Coefficient: 19.9; 95% CI = 12.7-27.0).

**Table 2:** Meta-regression effects of each empowerment domain on the countries’ Composite Coverage Index (CCI). (n = 62 countries)

	Crude		Adjusted*	
	Coefficient	95% CI	Coefficient	95% CI
Women’s empowerment:				
Attitude to violence	14.2	9.2-19.3	11.9	6.5-17.2
Social independence	16.1	11.1-21.1	16.3	9.3-23.3
Decision making	15.3	10.7-19.9	13.4	8.3-18.6

CI – confidence interval

*Adjusted by log Gross Domestic Product per capita (GDP adjusted by the Purchase Power Parity in International dollars).

**Figure 2:**
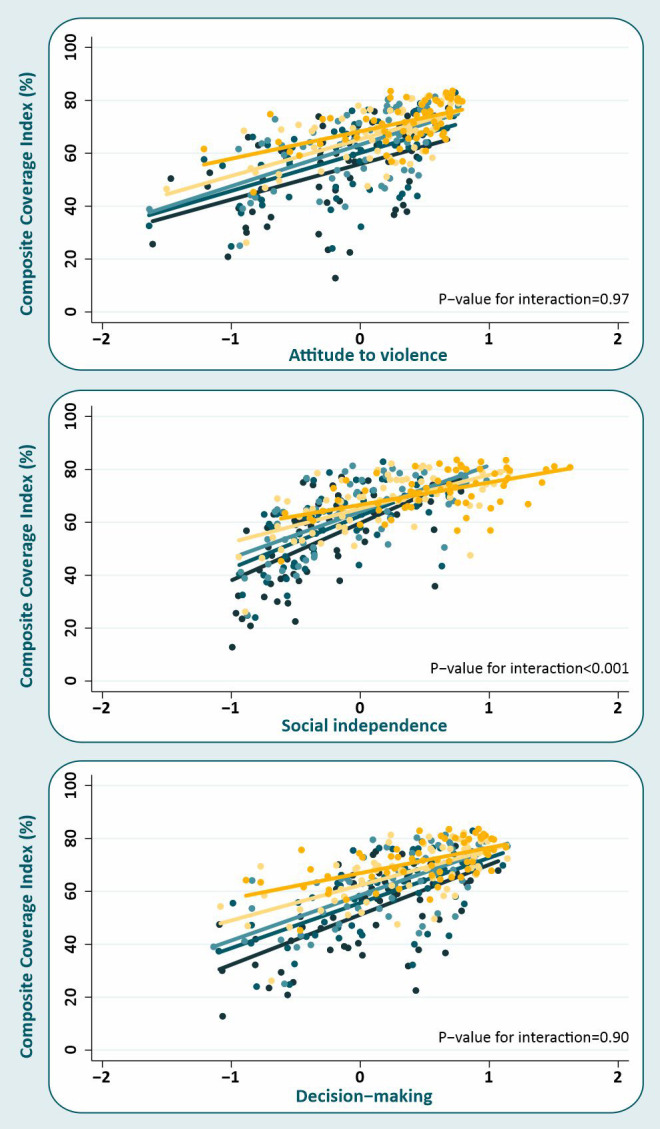
Effect of women’s empowerment for each of the three domains of the SWPER (attitude to violence, social independence and decision making) on the composite coverage index (CCI) for each of the five wealth quintiles.

We then performed stratified analyses by wealth quintiles to investigate whether the effect of the women’s empowerment on the CCI was modified by wealth. The CCI estimates and mean women’s empowerment level in attitude to violence, social independence and decision making by wealth quintiles are provided in Table S3 of the **Online Supplementary Document**. There was no evidence that the effects of attitude to violence and decision making changed with wealth (*P*-values for interaction were, respectively, 0.97 and 0.90) (**Figure 2**). In contrast, there was an important interaction between wealth and social independence (*P* < 0.001), with stronger associations among the poor (**Figure 2**). In the poorest quintile, one additional standard deviation in the social independence domain was associated with a 21.8 percentage points increase (95% CI = 14.0-29.6) in the CCI (**Table 3**). Among the richest, this effect was much smaller, at 8.7 percentage points (95% CI = 5.4-12.0).

**Table 3:** Meta-regression unadjusted effects of each empowerment domain on the countries’ Composite Coverage Index (CCI) by wealth quintile. (n = 62 countries)

	Women’s empowerment					
	Attitude to violence		Social independence		Decision making	
	Coeff	95% CI	Coeff	95% CI	Coeff	95% CI
Wealth quintiles:						
Poorest	13.4	6.7-20.1	21.8	14.0-29.6	18.9	12.7-25.1
2^nd^	14.4	8.6-20.2	20.0	13.6-26.4	17.1	11.6-22.6
3^rd^	15.7	10.7-20.8	17.6	12.5-22.7	17.1	12.4-21.8
4^th^	13.9	9.5-18.4	13.0	8.8-17.2	13.4	9.2-17.5
Wealthiest	10.6	6.6-14.5	8.7	5.4-12.0	9.7	6.3-13.2

CI – confidence interval

## DISCUSSION

We present a set of analyses of the associations between women’s empowerment and a composite measure of RMNCH intervention coverage. We found positive relationships between the three domains of women’s empowerment and the CCI at country level. The associations were statistically significant and programmatically relevant, which means that they should be taken into account when planning programs and strategies as empowering women is a desirable outcome in itself, and it can also improve RMNCH intervention coverage. One standard deviation increase in the attitude to violence, decision-making and social independence domains was associated with an increase of 14.2, 15.3 and 16.1 percentage points in CCI, respectively. The latter association was strongest in the poorest wealth quintile.

The social independence domain consists mainly of socio-demographic measures of life history (education, age at marriage, age at first birth, age and education gap with spouse) and by access to information. We were able to show that – even when the woman’s education was removed from the social independence score – the observed associations with coverage persisted. Such tangible measures may be more strongly associated with CCI because they reflect women’s agency more directly than the items in the decision-making and attitude to violence domains, which are more reflective of family and community norms, and as such, may constitute distal determinants in the processes that feed into intervention coverage. Even though decision-making is a key construct for agency while age at marriage or age at birth are proxies of it, such proxies may offer a more accurate representation of agency in these contexts. In other words, for LMIC women, particularly the poorest ones, life experiences (such as early marriage) may be a better means of assessing agency than perceived decision-making control. Besides that, our results for attitude to violence are in line with the literature that has shown that wife beating norms are related to actual partner violence [24], which in turn has been associated with compromised RMNCH [25,26].

We hypothesized that the effect of women’s empowerment on the CCI could be modified by wealth, potentially supporting women even in economically compromised circumstances. Economically vulnerable women experience greater restrictions in access to health resources and involvement in decision-making, relative to that experienced among the richer women [27]. The interaction with wealth was only significant for the social independence domain, which may be related to the lower variability of the average attitude to violence and decision-making scores across wealth quintiles than what is observed for social independence.

Our results show that the effect of social independence on the CCI was, on average, 2.5 times greater for the poorest women and children than for those in the richest quintile. Prior research has documented that national-level government effectiveness and political stability/absence of violence are also more strongly associated with RMNCH interventions among the poorest than the richest quintile [16]. The authors argued that the wealthier may benefit from safety nets that make them less dependent on state-provided services, which allows richer families to offset poor governance. The same argument may apply to our findings, suggesting that greater efforts to support gender-specific development and women’s social independence (eg, education, delayed marriage) may facilitate utilization of essential RMNCH health services amongst the poorest women and their children. This recommendation is in line with existing evidence [28-30] and growing calls from the field [18,31].

All the coverage indicators that comprise the CCI are based on maternal recall. Nondifferential recall would dilute the existing associations, however differential recall, which may happen if women have different recollections depending on their wealth or empowerment status, would lead to bias. One might expect that recall would be worse among the poorest, least-educated women, but the stronger associations with coverage in this group argue against this hypothesis. In addition, the surveys included in the analyses were conducted over 16 years and changes in gender relations might have occurred. However, it is known that such changes tend to be slow. The correlation between the year of the survey and the three empowerment domains were all between -0.11 and -0.02, thus ruling out this potential source of bias. Sensitivity analyses showed that by restricting the analyses to surveys conducted from 2010 to 2017, the association between women’s empowerment and the CCI was not changed for attitude to violence and decision making decision-making (Coefficient: 14.3; 95% CI = 8.6-20.0 and 16.4; 95% CI = 11.5-21.2, respectively) and was slightly stronger for social social independence (Coefficient: 18.3; 95% CI = 13.0-23.6).

Wealth quintiles are country-specific relative measures, and thus the poorest quintile in a middle-income country may be wealthier than say the second or third quintile in a poor country. Despite this limitation, use of wealth indices allows the systematic analyses of relative inequalities in health, using equal-sized categories, that would not be warranted with other measures of socioeconomic position [15]. Also, the strong association between the wealth index and most coverage indicators supports its effectiveness to discriminate subpopulations.

This is an ecological exploratory analysis, so we cannot infer causality from the results. The CCI is a summary measure of health service utilization coverage; the underlying availability of these services was not collected in the assessed data. One of the limitations of the SWPER is that it is restricted to partnered women (all three domains). However, most of the CCI components relate to maternal and child health. In the countries analyzed, on average 83.6% of the women who had one or more living children were married or in a union, ranging from 97.3% in Afghanistan to 45.9% in South Africa. Given the ecological design of this study, we believe analyses based on all women would not substantially impact the results. Also, summary measures such as the SWPER may mask differences in the individual indicators comprising each measure. Nonetheless, the advantages of the SWPER are substantial, including direct cross-country comparability [32]. In addition, the analyses based on three domains allow detecting which aspects of women’s empowerment are most strongly associated with RMNCH intervention coverage across countries. Identifying high and low performers opens avenues for policy makers to further investigate these gaps and initiate measures to accelerate progress towards the SDGs.

## CONCLUSIONS

Cross-national analysis shows strong associations between empowerment and the CCI in low- and middle-income countries, particularly in the area of women’s social independence. The poorest women and children are most affected by the association between disempowerment and RMNCH intervention coverage.

Empowering women is also a goal in itself as it gives them voice and agency to act upon their desires, so all countries should act to improve the women’s conditions. Our findings suggest that efforts towards reaching SDG5 (Achieving Gender Equality and Empowerment) may also have an important impact on health care utilization and therefore health outcomes in LMICs, given the demonstrated association between gender empowerment and coverage in this study. The magnitude of the coverage reported in our analyses has the capacity to result in substantial reductions in the number of deaths among women and children. However, changes in gender relations are difficult, slow processes, as they require changes in attitudes and practices that are undermined by contextual social norms [33,34]. More research is necessary to identify the best approaches to reduce gender inequalities and improve women’s empowerment in specific contexts. Our findings, jointly with the call for changes in gender relations issued by the SDGs, will give more prominence to this crucial issue and contribute towards reaching universal health coverage.

## Additional material

Online Supplementary Document
