# Supplementary material for: Is women’s empowerment associated with coverage of RMNCH interventions in low- and middle-income countries? An analysis using a survey-based empowerment indicator, the SWPER
Source: J Glob Health. 2021 Mar 1;11:04015. doi: 10.7189/jogh.11.04015 (PMC7979155; doi:10.7189/jogh.11.04015)
Supplement: Online Supplementary Document [file jogh-11-04015-s001.pdf]

Is women's empowerment associated with coverage of RMNCH interventions in low- and middle-income countries? An analysis using a survey-based empowerment indicator, the SWPER.

Fernanda Ewerling, Fernando C. Wehrmeister, Cesar G. Victora, Anita Raj, Lotus McDougal, Aluisio J. D. Barros

Online Supplementary Document

Table S1. Variables that compose the survey-based women's empowerment (SWPER) index and the changes made in the global version of the index (compared to the African-oriented SWPER index).

Item (v)	Code or unit	Changes
**Attitude to violence domain**		
1. Beating justified if wife goes out without telling husband	Yes = -1; DK=0; No=1	No changes
2. Beating justified if wife neglects the children	Yes = -1; DK=0; No=1	No changes
3. Beating justified if wife argues with husband	Yes = -1; DK=0; No=1	No changes
4. Beating justified if wife refuses to have sex with husband	Yes = -1; DK=0; No=1	No changes
5. Beating justified if wife burns the food	Yes = -1; DK=0; No=1	No changes
**Social independence domain**		
6. Frequency of reading newspaper or magazine	Not at all=0; <once a week=1; ≥once a week=2	No changes
7. Woman education in completed years of schooling	Years	No changes
8. Age of woman at first birth*	Years	No changes
9. Age at first cohabitation	Years	No changes
10. Age difference: woman's minus husband's age	Years	No changes
11. Education difference: woman's minus husband's years of schooling	Years	No changes
**Decision-making domain**		
12. Who usually decides on respondent's health care	Husband or other alone= -1; joint decision=0; respondent alone=1	**Husband or other alone= -1; Joint decision or respondent alone=1**
13. Who usually decides on large household purchases	Husband or other alone= -1; joint decision=0; respondent alone=1	**Husband or other alone= -1; Joint decision or respondent alone=1**
14. Who usually decides on visits to family or relatives	Husband or other alone= -1; joint decision=0; respondent alone=1	**Husband or other alone= -1; Joint decision or respondent alone=1**
X. Respondent worked in last 12 months	No = 0; In the past year = 1; Have a job, but on leave last 7 days = 2; Currently working = 2	**Item excluded**

* Following the SWPER methodology, age at first birth was imputed for those women who had not had a child.

Table S2. Definition of the Composite Coverage Index (CCI) indicators.

Intervention	Indicator definition	Numerator	Denominator
**Reproductive health**			
Demand for family planning satisfied with modern methods (DFPSm)	Percentage of women of reproductive age (15–49 years) who have their need for family planning satisfied with modern methods	Number of women of reproductive age (15–49 years) who have their need for family planning satisfied with modern methods	Total number of women of reproductive age (15–49 years) in need of family planning
**Maternal and newborn health**			
Antenatal care - four or more visits (ANC4)	Percentage of women attended four or more times during pregnancy by any provider	Number of women ages 15–49 who were attended four or more times during the pregnancy that led to their last birth in the 3 years preceding the survey by any provider	Total number of women ages 15–49 with a live birth in the 3 years preceding the survey
Skilled birth attendant (SBA)	Percentage of live births attended by skilled health personnel	Number of live births to women ages 15–49 years in the 3 years prior to the survey who were attended during delivery by skilled health personnel	Total number of live births to women ages 15–49 years in the 3 years preceding the survey
**Immunization**			
Immunized against Bacillus Calmette–Guérin (BCG)	Percentage of surviving infants who received BCG vaccine	Number of surviving infants who received BCG vaccine	Total number of surviving infants
Immunized with three doses of diphtheria–tetanus–pertussis (DPT3)	Percentage of infants who received three doses of diphtheria–tetanus–pertussis vaccine	Number of surviving infants who received three doses of diphtheria with tetanus toxoid and pertussis containing vaccine	Total number of surviving infants
Immunized against measles (MSL)	Percentage of surviving infants who received the first dose of measles containing vaccine	Number of surviving infants who received the first dose of measles containing vaccine by their first birthday (or as recommended in the national immunization schedule)	Total number of surviving infants
**Management of child illness**			
Oral rehydration salts treatment of diarrhoea (ORS)	Percentage of children ages 0–59 months with diarrhoea receiving oral rehydration salts (oral rehydration solution packets or pre-packaged oral rehydration solution fluids)	Number of children ages 0–59 months with diarrhoea in the two weeks prior to the survey receiving oral rehydration salts	Total number of children ages 0–59 months with diarrhoea in the two weeks prior to the survey

Careseeking for symptoms of pneumonia (CPNM)	Percentage of children ages 0–59 months with suspected pneumonia taken to a health care provider	Number of children ages 0–59 months with symptoms of pneumonia in the two weeks prior to the survey who were taken to a health care provider	Total number of children ages 0–59 months with symptoms of pneumonia (cough with fast breathing due to problem in the chest or problem in the chest and blocked nose) in the two weeks prior to the survey

Table S3. Composite Coverage Index estimate and mean women's empowerment level in attitude to violence, social independence and decision making by wealth quintiles.

Country	Survey year	CCI (%)					Mean women's empowerment														
							Attitude to violence					Social independence					Decision-making				
		Q1	Q2	Q3	Q4	Q5	Q1	Q2	Q3	Q4	Q5	Q1	Q2	Q3	Q4	Q5	Q1	Q2	Q3	Q4	Q5
Afghanistan	2015	39.2	43.4	43.3	52.8	60.6	-0.94	-0.80	-0.81	-0.81	-0.55	-0.58	-0.52	-0.48	-0.51	-0.37	-0.12	-0.35	-0.46	-0.31	-0.24
Albania	2008	57.2	63.8	66.8	66.3	66.9	-0.09	0.15	0.30	0.44	0.64	0.57	0.53	0.69	0.92	1.30	0.13	0.34	0.59	0.71	0.90
Angola	2015	22.5	32.2	46.8	60.8	71.1	-0.08	0.00	0.19	0.48	0.63	-0.50	-0.57	-0.36	-0.08	0.42	0.43	0.41	0.48	0.67	0.83
Armenia	2015	66.9	73.1	73.9	74.5	80.7	0.48	0.54	0.56	0.64	0.70	0.46	0.61	0.76	0.98	1.14	0.75	0.83	0.87	0.92	0.93
Azerbaijan	2006	35.8	43.5	50.5	47.5	56.9	-0.69	-0.55	-0.22	0.06	0.33	0.58	0.63	0.65	0.85	1.01	0.02	0.01	0.22	0.29	0.54
Bangladesh	2014	56.8	63.0	64.0	67.9	75.9	0.22	0.27	0.36	0.38	0.54	-0.88	-0.75	-0.66	-0.57	-0.11	0.01	0.07	0.11	0.12	0.28
Benin	2011	38.6	47.1	53.6	55.9	60.9	0.27	0.37	0.34	0.39	0.51	-0.62	-0.58	-0.51	-0.37	0.24	-0.09	-0.07	-0.02	0.05	0.30
Bolivia	2008	49.4	58.0	64.1	68.8	73.8	0.38	0.46	0.51	0.56	0.69	-0.18	0.00	0.21	0.50	1.11	0.61	0.74	0.79	0.86	0.99
Burkina Faso	2010	41.9	47.2	53.6	58.5	68.3	-0.16	-0.19	-0.18	-0.11	0.19	-0.78	-0.73	-0.72	-0.64	-0.17	-0.81	-0.84	-0.84	-0.74	-0.39
Burundi	2016	60.9	62.2	62.5	62.2	66.0	-0.61	-0.53	-0.44	-0.36	-0.03	-0.24	-0.16	-0.19	-0.14	0.37	0.22	0.27	0.27	0.38	0.55
Cambodia	2014	64.1	68.2	72.1	73.0	71.1	-0.29	-0.20	-0.17	-0.14	0.17	-0.03	0.03	0.10	0.18	0.46	0.85	0.86	0.88	0.88	0.87
Cameroon	2011	23.5	42.9	52.1	58.0	65.5	-0.25	-0.18	-0.10	0.07	0.37	-0.92	-0.59	-0.44	-0.12	0.35	-0.71	-0.42	-0.29	-0.12	0.09
Chad	2014	20.9	24.8	25.1	26.2	45.3	-1.03	-1.00	-0.93	-0.88	-0.83	-0.85	-0.88	-0.88	-0.89	-0.61	-0.56	-0.55	-0.59	-0.69	-0.47
Comoros	2012	41.3	48.4	54.3	60.8	56.9	-0.26	-0.06	0.03	0.13	0.24	-0.41	-0.36	-0.01	0.14	0.75	-0.54	-0.28	-0.10	-0.15	-0.11
Congo Democratic Republic	2013	37.9	42.3	45.6	52.0	59.1	-0.84	-0.76	-0.67	-0.63	-0.47	-0.48	-0.43	-0.42	-0.31	0.19	-0.23	-0.17	-0.19	0.01	0.12
Cote d'Ivoire	2011	29.4	38.6	41.7	53.1	59.1	-0.32	-0.23	-0.32	-0.30	0.20	-0.56	-0.57	-0.60	-0.45	0.00	-0.61	-0.56	-0.62	-0.47	-0.25
Dominican Republic	2013	77.1	82.9	77.8	79.8	79.6	0.68	0.74	0.76	0.77	0.79	-0.15	0.18	0.43	0.69	1.16	0.74	0.87	0.92	0.95	1.04
Egypt	2014	73.9	76.0	80.3	80.6	80.2	-0.32	-0.06	0.21	0.39	0.58	-0.21	-0.03	0.25	0.47	0.93	0.10	0.25	0.44	0.53	0.74
Eswatini	2006	70.1	75.5	79.5	81.3	82.9	0.31	0.41	0.52	0.52	0.67	-0.17	0.02	0.18	0.49	0.94	-0.17	-0.06	0.10	0.24	0.46
Ethiopia	2016	31.8	40.0	44.2	49.6	65.3	-0.89	-0.93	-0.85	-0.63	-0.12	-0.75	-0.75	-0.71	-0.61	-0.14	0.37	0.42	0.46	0.57	0.78

Gabon	2012	47.3	56.9	57.5	67.9	61.6	-0.32	-0.01	0.04	0.16	0.31	-0.17	-0.03	0.16	0.29	0.82	0.12	0.26	0.25	0.31	0.60
Gambia	2013	58.0	58.2	59.4	64.2	67.1	-0.57	-0.56	-0.59	-0.11	0.22	-0.72	-0.69	-0.64	-0.42	0.14	0.04	-0.01	0.07	0.01	0.21
Ghana	2014	60.7	61.1	68.0	68.5	69.8	-0.31	0.00	0.21	0.43	0.59	-0.51	-0.33	-0.16	0.29	0.86	0.26	0.52	0.59	0.51	0.55
Guatemala	2014	58.6	64.1	70.6	75.9	80.0	0.44	0.52	0.58	0.69	0.75	-0.38	-0.24	-0.03	0.32	0.82	0.27	0.49	0.65	0.81	0.94
Guinea	2012	25.6	32.6	38.8	46.5	61.6	-1.61	-1.64	-1.64	-1.50	-1.21	-0.96	-0.91	-0.90	-0.76	-0.39	-0.52	-0.51	-0.49	-0.55	-0.44
Guyana	2009	66.4	70.0	78.9	69.5	67.8	0.42	0.38	0.51	0.59	0.65	0.13	0.34	0.50	0.70	1.09	0.90	0.93	0.97	0.91	1.02
Haiti	2016	37.9	43.8	51.6	58.3	65.3	0.36	0.44	0.45	0.56	0.66	-0.16	-0.01	0.22	0.42	1.01	0.45	0.52	0.60	0.65	0.71
Honduras	2011	74.1	78.8	81.3	82.2	83.6	0.37	0.46	0.58	0.66	0.72	-0.37	-0.24	-0.06	0.24	0.75	0.32	0.48	0.66	0.80	0.92
India	2015	59.9	68.8	74.4	78.0	81.0	-0.19	-0.14	-0.10	-0.02	0.23	-0.43	-0.30	-0.08	0.21	0.79	0.28	0.35	0.40	0.49	0.63
Indonesia	2012	63.0	76.3	78.6	80.7	78.4	0.13	0.24	0.30	0.40	0.48	0.01	0.17	0.31	0.53	1.04	0.56	0.62	0.69	0.71	0.80
Kenya	2014	56.4	67.2	71.4	76.2	80.0	-0.31	-0.10	-0.01	0.23	0.50	-0.47	-0.12	-0.02	0.23	0.68	0.15	0.38	0.44	0.55	0.66
Kyrgyzstan	2012	71.7	70.9	68.1	66.3	75.7	0.09	-0.04	-0.03	0.22	0.50	0.78	0.68	0.72	0.85	1.13	0.82	0.79	0.72	0.90	1.03
Lesotho	2014	70.5	72.0	77.5	76.1	79.8	-0.02	0.14	0.28	0.33	0.60	0.00	0.10	0.20	0.28	0.74	0.42	0.58	0.60	0.70	0.84
Liberia	2013	50.6	59.4	63.9	65.0	64.6	-0.08	-0.06	-0.13	0.09	0.20	-0.71	-0.71	-0.60	-0.37	0.14	0.48	0.45	0.47	0.56	0.62
Madagascar	2008	36.7	43.2	50.5	58.3	68.3	0.26	0.30	0.23	0.23	0.33	-0.69	-0.50	-0.36	-0.17	0.45	0.66	0.69	0.74	0.74	0.83
Malawi	2015	75.1	76.4	76.7	78.6	78.6	0.42	0.45	0.46	0.48	0.62	-0.44	-0.42	-0.42	-0.33	0.13	0.12	0.15	0.21	0.22	0.48
Maldives	2009	75.8	76.2	72.5	75.4	67.4	0.11	0.20	0.22	0.32	0.54	0.06	0.20	0.33	0.47	0.82	0.41	0.45	0.51	0.56	0.62
Mali	2012	30.0	37.4	39.1	54.3	64.2	-0.88	-0.92	-0.91	-0.85	-0.63	-0.64	-0.73	-0.72	-0.68	-0.29	-1.07	-1.10	-1.14	-1.08	-0.89
Moldova	2005	67.9	69.8	77.1	72.4	76.7	0.18	0.36	0.52	0.61	0.69	0.55	0.69	0.81	0.95	1.14	1.07	1.11	1.14	1.14	1.13
Morocco	2003	50.4	57.7	63.7	68.3	72.3	-1.47	-1.21	-0.76	-0.38	0.11	-0.38	-0.30	-0.16	0.15	0.62	-0.46	-0.37	-0.08	0.11	0.40
Mozambique	2011	40.4	44.6	53.4	62.1	72.0	0.33	0.33	0.40	0.42	0.57	-0.52	-0.51	-0.58	-0.47	0.04	-0.02	0.00	0.15	0.24	0.60
Myanmar	2015	56.8	62.7	68.2	76.6	83.5	-0.04	-0.03	-0.03	0.08	0.24	0.04	0.24	0.46	0.57	1.13	0.46	0.58	0.59	0.64	0.69
Namibia	2013	70.3	76.4	77.9	78.8	81.1	-0.29	0.06	0.21	0.48	0.68	0.11	0.46	0.78	0.95	1.50	0.55	0.56	0.70	0.83	0.97
Nepal	2016	60.9	62.2	64.7	68.3	68.7	0.40	0.38	0.28	0.39	0.51	-0.45	-0.38	-0.42	-0.26	0.30	-0.19	-0.15	-0.15	-0.04	0.27
Nicaragua	2001	63.6	75.9	79.2	79.8	82.2	0.29	0.42	0.53	0.63	0.71	-0.61	-0.36	-0.12	0.13	0.61	0.19	0.41	0.55	0.76	0.89
Niger	2012	32.2	40.4	41.2	46.9	63.4	-0.72	-0.76	-0.85	-0.74	-0.31	-0.95	-0.94	-0.93	-0.94	-0.58	-0.81	-0.85	-0.90	-0.87	-0.77
Nigeria	2013	12.8	24.0	39.8	51.1	67.4	-0.19	-0.22	-0.12	0.20	0.47	-0.99	-0.81	-0.50	-0.05	0.76	-1.07	-0.80	-0.38	-0.09	0.33
Pakistan	2017	46.4	55.8	63.3	69.9	74.4	-0.77	-0.55	-0.16	0.13	0.44	-0.44	-0.34	-0.11	0.25	0.69	-0.45	-0.48	-0.34	-0.19	-0.01

Peru	2016	65.3	74.1	75.1	79.0	80.8	0.66	0.72	0.75	0.76	0.76	-0.01	0.44	0.82	1.09	1.63	0.37	0.73	0.87	0.94	0.95
Philippines	2017	62.9	70.8	74.0	74.5	75.1	0.53	0.61	0.65	0.68	0.70	0.28	0.56	0.78	1.04	1.41	0.82	0.91	0.90	0.97	0.99
Rwanda	2014	63.8	67.9	68.7	68.9	71.4	-0.09	-0.03	0.08	0.12	0.41	0.10	0.19	0.21	0.34	0.70	0.37	0.44	0.54	0.53	0.69
Sao Tome & Principe	2008	66.9	63.3	67.4	69.5	74.4	0.34	0.44	0.48	0.38	0.51	-0.32	-0.32	-0.15	-0.09	0.40	0.15	0.13	0.34	0.25	0.45
Senegal	2017	47.5	55.6	64.1	69.5	75.7	-1.18	-0.77	-0.39	-0.06	0.29	-0.69	-0.47	-0.28	0.00	0.60	-1.09	-0.99	-0.84	-0.77	-0.46
Sierra Leone	2013	63.0	63.1	64.8	69.0	72.9	-0.79	-0.72	-0.72	-0.61	-0.29	-0.67	-0.65	-0.64	-0.64	-0.21	-0.05	-0.18	-0.10	-0.02	0.02
South Africa	2016	74.1	69.5	75.8	78.9	79.9	0.58	0.67	0.69	0.70	0.75	0.56	0.69	0.98	1.16	1.44	0.84	0.85	0.95	0.99	1.02
Tajikistan	2012	66.1	66.2	72.8	70.7	73.2	-0.87	-0.83	-0.66	-0.48	-0.23	0.43	0.44	0.51	0.59	0.74	-0.06	-0.15	0.03	0.11	0.24
Tanzania	2015	50.1	57.0	62.9	69.6	72.9	-0.76	-0.55	-0.61	-0.35	0.04	-0.36	-0.29	-0.14	0.02	0.51	-0.21	-0.05	0.02	0.14	0.29
Timor-Leste	2016	52.6	55.3	61.6	68.5	74.8	-1.17	-1.07	-0.94	-0.90	-0.70	0.03	0.13	0.23	0.43	0.90	0.79	0.81	0.83	0.92	0.98
Togo	2013	46.0	46.2	47.6	56.4	65.7	-0.19	0.07	0.16	0.27	0.44	-0.57	-0.44	-0.36	-0.12	0.40	-0.31	-0.17	-0.16	-0.20	-0.25
Uganda	2016	59.1	62.0	65.0	67.9	71.7	-0.31	-0.19	-0.05	0.04	0.35	-0.50	-0.44	-0.35	-0.15	0.39	0.29	0.21	0.25	0.33	0.49
Zambia	2013	61.1	65.3	67.8	75.9	81.2	-0.57	-0.51	-0.36	0.03	0.36	-0.47	-0.41	-0.32	-0.14	0.43	0.09	0.19	0.30	0.55	0.80
Zimbabwe	2015	66.4	68.2	76.0	76.4	81.7	-0.04	0.14	0.20	0.33	0.54	-0.18	-0.15	0.01	0.36	0.78	0.60	0.65	0.71	0.83	0.96
